# Variant insertion of coracobrachialis muscle in a South Karnataka cadaver

**DOI:** 10.1186/1757-1626-1-291

**Published:** 2008-10-31

**Authors:** Bhagath K Potu, Muddanna S Rao, Soubhagya R Nayak, Venkata R Vollala, Anil K Mandava, Huban Thomas

**Affiliations:** 1 Department of Anatomy, Centre for Basic Sciences, Kasturba Medical College, Manipal University, Manipal, Karnataka, India; 2 Department of Anatomy, Centre for Basic Sciences, Kasturba Medical College, Manipal University, Mangalore, Karnataka, India; 3 Department of Anatomy, Melaka Manipal Medical College (Manipal Campus), Manipal University, Manipal, Karnataka, India; 4 Department of Orthopaedic Surgery, Kasturba Hospital, Manipal University, Manipal, Karnataka, India

## Abstract

**Background:**

The coracobrachialis is a muscle of arm has been known for its morphological variations. The variation of the coracobrachialis reported in this case is unique and to the best of our knowledge this variation is not reported in south karnataka population.

**Case presentation:**

During routine dissection, we observed an unusual insertion of a coracobrachialis muscle with an accessory slip in the right arm of an old male cadaver. It extended from the superficial fibres of coracobrachialis downwards and medially in front of the median nerve and brachial artery and finally inserted on anteromedial aspect of the medial epicondyle.

**Conclusion:**

The existence of abnormal insertion of the coracobrachialis muscle presented in this case should be kept in mind in a patient presenting with high median nerve palsy together with symptoms of brachial artery compression.

## Background

Coracobrachialis muscle is classically mentioned as taking its origin from the tip of the coracoid process of the scapula, where it is blended with the medial side of the short head of biceps brachii. The tendon is inserted into the medial border of the shaft of the humerus, where the nutrient foramen is located. The musculocutaneous nerve usually pierces the coracobrachialis muscle in man [1,2].

Morphologic variations of the Coracobrachialis muscle have been known for a long time [3,4] and include accessory slips that attach to the lesser tubercle, medial supracondylar ridge, medial intermuscular septum. This study describes a variation of the coracobrachialis muscle that crossed anterior to the median nerve and brachial artery in the right arm of an old male cadaver.

## Case presentation

**Figure 1:**
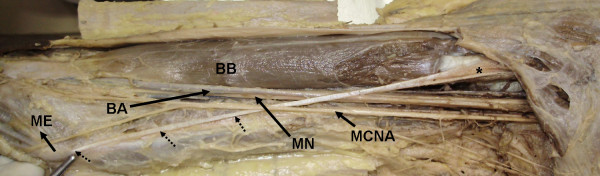
**A photograph of the right arm of an old male cadaver demonstrating insertion of an accessory slip of the coracobrachialis muscle as a slender tendon (Dashed lines)**. Notice that: the tendon passing inferiomedially crossing anterior to the Median Nerve (MN) and Brachial artery (BB) and attached to the medial epicondyle (ME) of the humerus. * showing the proximal portion of anomalous tendon. We can also see Biceps brachii muscle (BB) and Medial cutaneous nerve of arm (MCNA) in the picture.

During routine anatomical dissection in our department, we observed an unusual insertion of a coracobrachialis muscle with an accessory slip in the right arm of an old male cadaver. It extended from the superficial fibres of coracobrachialis downwards and medially in front of the median nerve and brachial artery and finally inserted on anteromedial aspect of the medial epicondyle (Fig. 1). The length of the part described is 20 cms and the diameter is 0.2 cms.

## Discussion

The clinical implication of the accessory slip of coracobrachialis is that it has the potential to cause median nerve entrapment and brachial artery compression. Although we could not detect similar cases described in the available literature, other muscular abnormalities were described, which may lead to a comparable arrangement and injury. Various studies have described the compression of median nerve and brachial artery with anomalous muscles [5-9]. The existence of abnormal insertion of the coracobrachialis muscle should be kept in mind in a patient presenting with high median nerve palsy together with symptoms of brachial artery compression. It may lead to wasting or ischaemic contraction of flexors of the forearm. This variation is important to note during the active use of coracobrachialis as a transposition flap in deformities of infraclavicular and axillary areas and in postmastectomy reconstruction [10], during surgical intervention of the anterior compartment of the arm, such as trauma, tumour, neurovascular disease; while using coracobrachialis as a vascularized muscle for transfer for the treatment of longstanding facial paralysis [11].

The morphological variations of the coracobrachialis muscle may be due to failure of muscle primordia to disappear during embryological development [12] and this might be a reason for the presence of an unusual lengthy accessory insertion of the coracobrachialis muscle reported in this case.

## Competing interests

The authors declare that they have no competing interests.

## Authors' contributions

BKP did the literature search and wrote the case report and also obtained written consent. MSR conceived the study and helped to draft the manuscript. SRN, VRV, AKM and HT helped in the literature search. All authors had gone through the final manuscript and approved it.

## Consent

Written informed consent was obtained from the subject's relative for publication of this case report.
